# Regulating Drug Release Performance of Acid-Triggered Dimeric Prodrug-Based Drug Self-Delivery System by Altering Its Aggregation Structure

**DOI:** 10.3390/molecules29153619

**Published:** 2024-07-31

**Authors:** Chen Yang, Peng Liu

**Affiliations:** State Key Laboratory of Applied Organic Chemistry, College of Chemistry and Chemical Engineering, Lanzhou University, Lanzhou 730000, China; yangch21@lzu.edu.cn

**Keywords:** tumor chemotherapy, dimeric prodrug, aggregation structure, acid-triggered, doxorubicin

## Abstract

Dimeric prodrugs have been investigated intensely as carrier-free drug self-delivery systems (DSDSs) in recent decades, and their stimuli-responsive drug release has usually been controlled by the conjugations between the drug molecules, including the stimuli (pH or redox) and responsive sensitivity. Here, an acid-triggered dimeric prodrug of doxorubicin (DOX) was synthesized by conjugating two DOX molecules with an acid-labile ketal linker. It possessed high drug content near the pure drug, while the premature drug leakage in blood circulation was efficiently suppressed. Furthermore, its aggregation structures were controlled by fabricating nanomedicines via different approaches, such as fast precipitation and slow self-assembly, to regulate the drug release performance. Such findings are expected to enable better anti-tumor efficacy with the desired drug release rate, beyond the molecular structure of the dimeric prodrug.

## 1. Introduction

Drug delivery systems (DDSs) have been investigated intensely in recent decades, with the aim of improving the antitumor efficacy of chemotherapeutic drugs [1,2] by enhancing their solubility and bioavailability but minimizing their toxicity and side effects on the normal cells and tissue. The carrier-mediated approach was the earliest proposed strategy, in which the chemotherapeutic drug was non-covalently loaded into the carrier [3] or covalently conjugated onto the carrier via various stimuli-responsive dynamic covalent bonds as carrier-mediated prodrugs, which could be cleaved off with exogenous stimuli (temperature, light, or magnetic field) or endogenous stimuli (pH, glutathione (GSH), reactive oxygen species (ROS), or specific enzymes) [4,5,6,7] to release the drug. Owing to the covalent conjugation via dynamic covalent bonds, better drug release performance could be achieved with the carrier-mediated prodrugs, showing higher antitumor efficacy, lower toxicity, and fewer side effects of the desired intracellular drug release, with minimized premature drug leakage during blood circulation.

However, the drug content in the carrier-mediated DDSs, including carrier-mediated prodrugs, is usually low, due to the carriers. This means that a higher dose or higher administration frequency is needed in their practical application. Furthermore, the cytotoxicity and immunogenicity of the carriers should be considered in their design and development [8].

Recently, carrier-free drug self-delivery systems (DSDSs), in which no carrier is needed, have attracted more attention [9,10,11]. Using these systems, the possible cytotoxicity and immunogenicity of the carriers can be avoided. Most importantly, high drug content is obtained near to the pure drugs. Among these systems, dimeric prodrugs, via conjugating two drug molecules with one dynamic covalent bond, have been recognized as a promising kind of DSDS [12]. Covalent dynamic conjugation can not only minimize the premature drug leakage during blood circulation by reducing the drug’s solubility, but also release the drug in response to specific intracellular stimuli, such as pH [12,13,14,15,16,17], GSH [13,18,19,20,21], ROS [13,22], pH/GSH [23], or redox (GSH/ROS) [24,25,26,27,28,29,30,31,32], achieving on-demand intracellular drug release. Moreover, two different drugs can be conjugated for tumor combination chemotherapy [33,34]. Besides investigations of the molecular structures of the dimeric prodrugs, the aggregation structure has also been recently explored as another important factor impacting their drug release and antitumor efficacy [21,23].

Doxorubicin (DOX) is a broad-spectrum antitumor drug, which strikingly hinders the growth of tumor cells in various cellular growth cycles by inhibiting the synthesis of RNA and DNA [35]. Dimerization of DOX can efficiently avoid premature drug leakage in blood circulation and minimize its toxic side effects. Furthermore, cell-targeted drug release can be achieved for better antitumor efficacy, by coupling with pH- [13,14,15,16,17], GSH- [13,18,20,21], ROS- [13,22], pH/GSH- [23], or redox-triggered dynamic covalent bonds [32]. Additionally, drug release is controlled by the stability of the used dynamic covalent bonds. In pH-triggered dimeric prodrugs, the hydrazone-mediated dimer exhibited a faster acid-triggered DOX release with a higher premature DOX leakage, due to the high hydrolysis activity [14]. Due to the high stability of the amide and carbamate groups, the amide- and carbamate-mediated dimers showed slow acid-triggered DOX release, despite negligible premature DOX leakage [15,16].

**Scheme 1:**
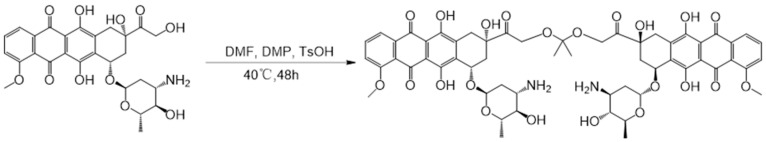
Synthesis of the ketal-mediated dimeric prodrug of DOX (DDOX_ketal_) by coupling with 2,2-dimethoxypropane (DMP).

As a pH-sensitive hydrolysable linkage with a hydrolysis rate that is increased 10 times with each unit of pH decrease [36], ketal has been widely used in the design of drug carriers for pH-triggered release [37,38]. Recently, it has also been utilized in antitumor prodrugs [39,40,41,42,43,44,45]. At present, there is no report on the ketal-mediated prodrug of DOX. Here, the ketal-mediated dimeric prodrug of DOX (DDOX_ketal_) was designed (Scheme 1). Furthermore, DDOX_ketal_ was assembled as acid-triggered nanoparticles with different fabrication approaches to investigate the effect of the aggregation structures of the dimers on their drug release and in vitro antitumor efficacy.

## 2. Results

### 2.1. Synthesis and Characterization of Ketal-Bridged Doxorubicin Dimeric Prodrug (DDOX_ketal_)

**Figure 1:**
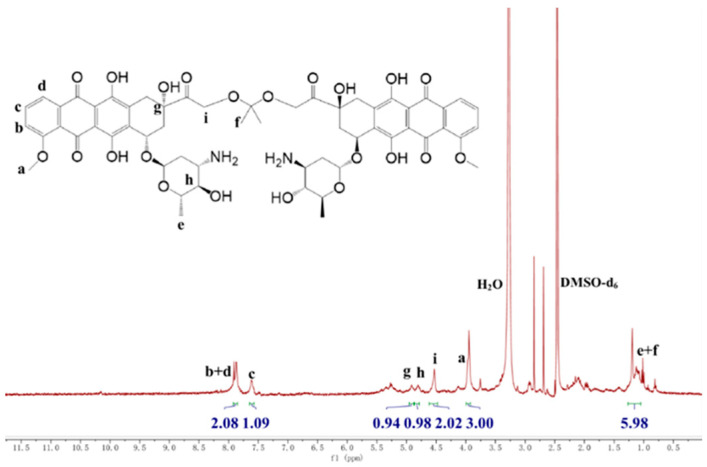
^1^H NMR spectrum of the proposed ketal-bridged doxorubicin dimeric prodrug (DDOX_ketal_).

The acid-sensitive ketal-bridged doxorubicin dimeric prodrug (DDOX_ketal_) was synthesized by coupling two DOX molecules with an acid-sensitive acetone-based ketal conjugation (Scheme 1). Besides the characteristic proton signals on DOX of H_a_ (δ = 3.92–3.99 ppm, 3.00 H), H_b+d_ (δ = 7.83–7.91 ppm, 2.08 H), H_c_ (δ= 7.57–7.65 ppm, 1.09 H), H_g_ (δ = 4.87–4.96 ppm, 0.94 H), and H_h_ (δ = 4.78–4.87 ppm, 0.98 H), H_i_ shifted from δ = 4.77 ppm to δ = 4.48–4.62 ppm (2.02 H), indicating the coupling on the C-14 position (Figure 1) [46]. Although the proton signals of the methyl on DOX (H_e_) and the methyl on ketal linkage (H_f_) were overlapped at δ = 1.05–1.26 ppm (5.98 H), the integral area ratio between the methoxy on DOX (H_a_) and the methyl (H_e+f_) was found to be 3.00:5.98, which was very near to the theoretical value of 1:2, indicating the successful synthesis of the ketal-bridged doxorubicin dimeric prodrug (DDOX_ketal_), as illustrated in Scheme 1.

Because the coupling reaction was not on the chromophore of DOX, anthraquinone, the proposed ketal-bridged doxorubicin dimeric prodrug (DDOX_ketal_) showed the identical UV-vis absorption as DOX in dimethylsulfoxide (DMSO) solution at the same equivalent DOX molar concentration. So, the DOX content in the ketal-bridged doxorubicin dimeric prodrug (DDOX_ketal_) could be measured by determining its DOX molar content by UV-vis absorption at 480 nm and calculated with the calibration curve of DOX in DMSO solution. It was determined to be 1.71 × 10^−3^ mmol/g, very near to the theoretical value of 1.77 × 10^−3^ mmol/g. This result also demonstrated the successful synthesis of ketal-bridged doxorubicin dimeric prodrug (DDOX_ketal_).

**Figure 2:**
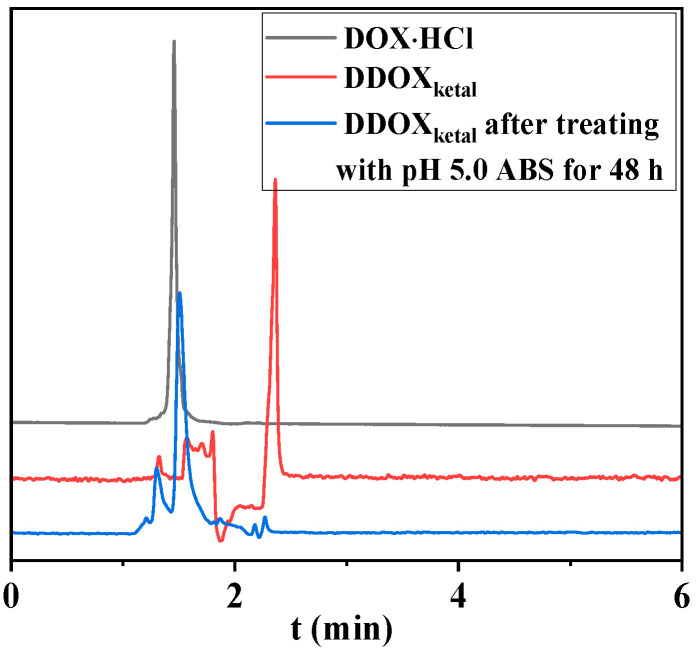
HPLC results of DOX and the proposed DDOX_ketal_ before and after treatment with pH 5.0 ABS for 48 h.

To reveal the acid-triggered cleavage of the proposed ketal-bridged doxorubicin dimeric prodrug (DDOX_ketal_), it was treated with pH 5.0 acetate buffer solution (ABS) at 37 °C for 48 h. After centrifugation (10^4^ rpm for 10 min), the solution was analyzed by the high-performance liquid chromatography (HPLC) technique, with acetonitrile/water (vol: 3/7) containing 0.1% acetic acid as mobile phase. Because the dimeric prodrug could not be dissolved in the pH 5.0 ABS, the remained dimeric prodrug was removed by centrifugation. Therefore, there was no signal of the dimeric prodrug at 2.36 min (Figure 2), and the solution gave only one signal at the elution time of 1.51 min, which was slightly different from the signal of DOX⋅HCl at 1.46 min due to the different protonation degrees. Such results demonstrated that the acetone-based ketal conjugation could be cleaved off with pH 5.0 ABS as a self-immolative linker to release the parent drug (DOX) [47], indicating a desired acid-triggered drug release in the endosome–lysosome system [48].

### 2.2. Fabrication and Characterization of DDOX_ketal_ Nanoparticles

**Figure 3:**
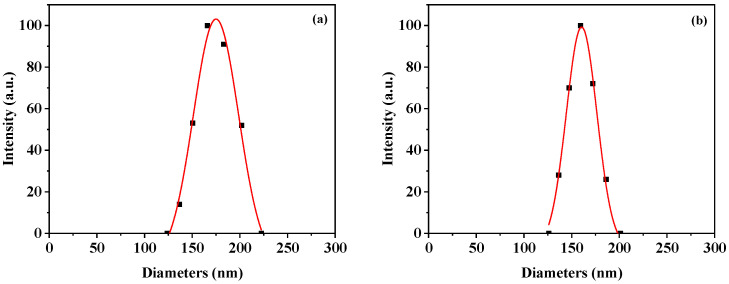
Typical hydrodynamic diameter and distribution of the DDOX_ketal_-NPs1 nanoparticles (**a**) and the DDOX_ketal_-NPs2 nanoparticles (**b**).

To investigate the effect of the aggregation structures on their acid-triggered drug release and in vitro cytotoxicity, the DDOX_ketal_ nanoparticles were fabricated via two different approaches: DDOX_ketal_-NPs1 nanoparticles via dropping their *N,N*-dimethylformamide (DMF) solution into bad solvent (water), and DDOX_ketal_-NPs2 nanoparticles via dropping water into their DMF solution. In the dynamic light scattering (DLS) analysis, with the same concentration (1.0 mg/mL) in DMF solution, DMF/water ratio, and dropping rate, a slightly bigger mean hydrodynamic diameter (D_h_) of 172.2 ± 28.5 nm (PDI = 0.0275) was achieved with the DDOX_ketal_-NPs1 nanoparticles via dropping their DMF solution into water, than with the DDOX_ketal_-NPs1 nanoparticles via dropping water into their DMF solution (161.5 nm ± 21.7 nm (PDI = 0.0181)), despite a similar narrow normal distribution (Figure 3).

**Figure 4:**
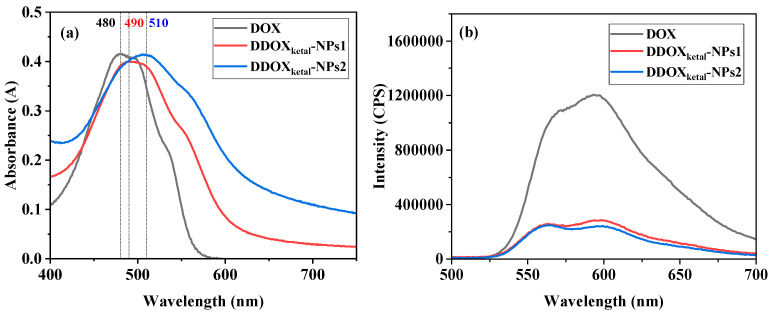
UV-vis (**a**) and fluorescence (**b**) spectra of aqueous DOX solution and the aqueous dispersions of the DDOX_ketal_-NPs1 nanoparticles and the DDOX_ketal_-NPs2 nanoparticles (at the same equivalent DOX concentration of 40 μg/mL).

UV-vis and fluorescence analysis were used to investigate the driving force in the fabrication of the DDOX_ketal_ nanoparticles. In the UV-vis spectra (Figure 4a), the maximum absorption of the DDOX_ketal_ nanoparticles shifted from 480 nm for DOX, to 490 nm and 510 nm for the DDOX_ketal_-NPs1 nanoparticles and the DDOX_ketal_-NPs2 nanoparticles. Furthermore, the fluorescence of DOX was significantly quenched in the dimeric prodrug nanoparticles, especially the DDOX_ketal_-NPs2 nanoparticles (Figure 4b). The red-shift in UV-vis absorption and the fluorescence quenching indicated the π-π stacking interaction between the DOX units in the dimeric prodrugs [49,50,51,52]. The red-shift in UV-vis absorption and the fluorescence quenching were greater in the DDOX_ketal_-NPs2 nanoparticles, meaning a compacter aggregation structure was achieved via the slow assembly by dropping water into the DMF solution [21,23].

**Figure 5:**
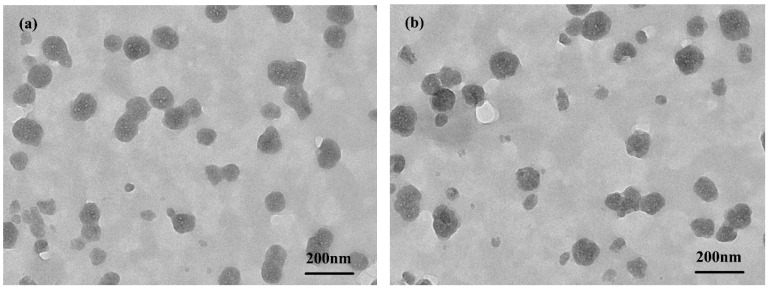
TEM images of the DDOX_ketal_-NPs1 nanoparticles (**a**) and the DDOX_ketal_-NPs2 nanoparticles (**b**).

In transmission electron microscope (TEM) observations (Figure 5), similar near-spherical nanoparticles could be seen for the DDOX_ketal_-NPs1 nanoparticles and the DDOX_ketal_-NPs2 nanoparticles, with average particle sizes of 145.8 nm and 151.0 nm, respectively. Compared with the DDOX_ketal_-NPs2 nanoparticles, the DDOX_ketal_-NPs1 nanoparticles showed a bigger Dh in the DLS analysis but a smaller particle size in the TEM analysis, due to a higher swelling degree in water. Interestingly, the mesopores around 8 nm could be seen in both nanoparticles (the regions with a lower contrast ratio in the nanoparticles), distinctly different from the reported DOX-based dimeric prodrug nanoparticles via various linkers [13,14,15,16,17,18,20,21,22,23,32]. The difference likely results from the flexible linker length between the rigid framework on DOX. The linker in the ketal-bridged doxorubicin dimeric prodrug (DDOX_ketal_) was the shortest, and restricts the molecular deformation. As a result, the π-π stacking interaction between the DOX units in different dimeric prodrugs was impeded, and a much looser aggregation structure was obtained for the proposed ketal-bridged doxorubicin dimeric prodrug (DDOX_ketal_).

The ketal-bridged doxorubicin dimeric prodrug (DDOX_ketal_) was designed by coupling two DOX molecules at their C-14 position (Scheme 1). The amino group on DOX was retained, making a hydrophilic surface. Thus, the DDOX_ketal_ nanoparticles could be swollen in an aqueous system. Moreover, because a looser aggregation structure resulted from the lower degree of π-π stacking interaction, a higher swelling degree was caused in the DDOX_ketal_-NPs1 nanoparticles. In general, the porous structure and hydrophilic surface of both DDOX_ketal_ nanoparticles are expected to accelerate the drug release in an acidic intracellular microenvironment, via the protonation of the surface amino groups and effortless diffusion of H^+^ ions into the pores of the dimeric prodrug nanoparticles, which attack the acid-labile ketal conjugation by nucleophiles (potentially water) after protonation.

### 2.3. In Vitro Acid-Triggered Drug Release

**Figure 6:**
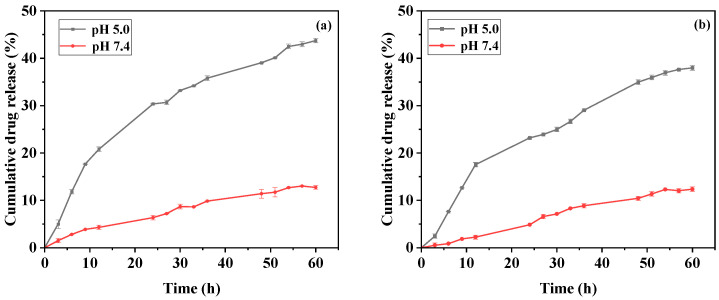
Acid-triggered drug release from the DDOX_ketal_-NPs1 nanoparticles (**a**) and the DDOX_ketal_-NPs2 nanoparticles (**b**) in releasing media with different pH values.

The acid-triggered drug release from the proposed DDOX_ketal_ nanoparticles was then assessed in vitro in releasing media with different pH values, using pH 7.4 phosphate buffered saline (PBS) and pH 5.0 acetate buffered solution (ABS), mimicking the acidity of the blood and endosome–lysosome system, respectively. Both the DDOX_ketal_-NPs1 nanoparticles and the DDOX_ketal_-NPs2 nanoparticles showed sustained drug release without any boosting release (Figure 6). The cumulative DOX release reached 43.76 ± 0.50% and 37.98 ± 0.54% at pH 5.0 ABS within 60 h for the DDOX_ketal_-NPs1 nanoparticles and the DDOX_ketal_-NPs2 nanoparticles, while the premature drug leakage was 12.75 ± 0.36% and 12.37 ± 0.47% at pH 7.4 PBS.

The DDOX_ketal_-NPs1 nanoparticles possessed a higher cumulative DOX release at pH 5.0 ABS and higher premature DOX leakage at pH 7.4 PBS than the DDOX_ketal_-NPs2 nanoparticles. This is likely caused by fewer π-π stacking interactions and the looser aggregation structure of the DDOX_ketal_-NPs1 nanoparticles [21,23]. After the acid cleavage of the ketal conjugation, the DOX molecules were protonated and released easily from the surfaces of the nanoparticles by dissolving.

### 2.4. In Vitro Cellular Uptake and Cytotoxicity

**Figure 7:**
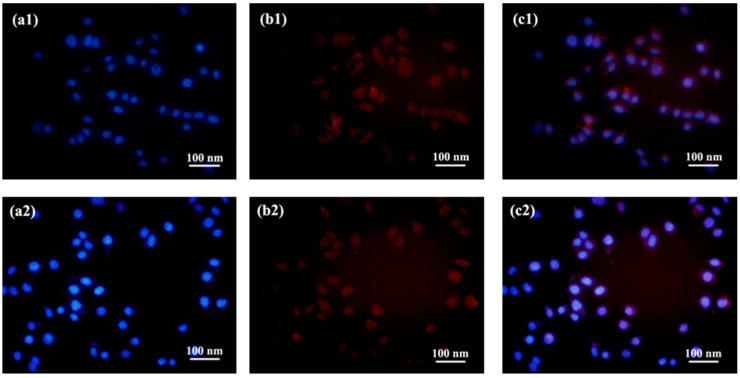
CLSM images of the HepG2 cells after incubation with the DDOX_ketal_-NPs1 nanoparticles (1) and the DDOX_ketal_-NPs2 nanoparticles (2) at a dose of 15 μg/mL for 48 h: DAPI (**a**), DOX (**b**), and merged (**c**).

To assess the in vitro cytotoxicity of the DDOX_ketal_ nanoparticles and investigate the effect of the aggregation structure on the antitumor efficacy, the in vitro cellular uptake of DDOX_ketal_ nanoparticles was studied with the confocal laser scanning microscope (CLSM) technique, after incubating liver cancer cells (HepG2) in the presence of 15 µg/mL of the DDOX_ketal_ nanoparticles at 37 °C for 48 h. After cellular fixing, nuclei staining, and thorough washing, the red fluorescence of DOX could be seen in the HepG2 cells (Figure 7), revealing the successful internalization of the DDOX_ketal_-NPs1 nanoparticles and the DDOX_ketal_-NPs2 nanoparticles by the HepG2 cells.

**Figure 8:**
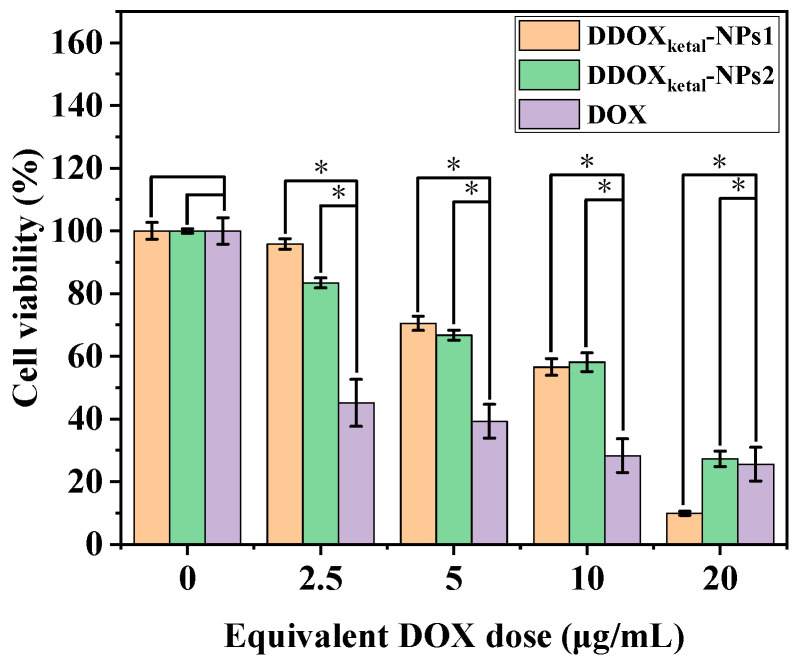
In vitro cytotoxicity of the free DOX and DDOX_ketal_ nanoparticles on HepG2 cells after incubation for 48 h. The data are given as the mean ± S.D. (n = 3) (* denotes significant difference *p* < 0.05).

Moreover, the cytotoxicity of the DDOX_ketal_-NPs1 nanoparticles and DDOX_ketal_-NPs2 nanoparticles on the HepG2 cells was assessed via the 3-(4,5-dimethylthiazol-2-yl)-2,5-diphenyltetrazolium bromide (MTT) assay after incubation in the presence of the nanomedicines for 48 h. Both nanomedicines showed dose-dependent cytotoxicity on the HepG2 cells as free DOX (Figure 8). Higher cell viability was obtained with the DDOX_ketal_ nanoparticles than free DOX at the lower DOX-equivalent dose (≤10 μg/mL), which may be due to the slow DOX release from the DDOX_ketal_ nanoparticles. However, a much lower cell viability of 9.9% was obtained with the DDOX_ketal_-NPs1 nanoparticles at a DOX-equivalent dose of 20 μg/mL, than with the DDOX_ketal_-NPs2 nanoparticles and free DOX. Such results probably resulted from the P-glycoprotein (P-gp) inhibition via the nanomedicines [53], where the nanocarrier systems are internalized via endocytosis or phagocytosis while the free drug is internalized via direct diffusion. The half-maximal inhibitory concentration (IC50) was calculated to be 11.13 μg/mL, 12.54 μg/mL, and 8.51 μg/mL for the DDOX_ketal_-NPs1 nanoparticles, DDOX_ketal_-NPs2 nanoparticles, and free DOX, respectively, calculated with the DFT-based Computational Methodology of IC50 Prediction.

Furthermore, it was found that the in vitro cytotoxicity of the DDOX_ketal_-NPs1 nanoparticles on the HepG2 cells was lower than that of the DDOX_ketal_-NPs2 nanoparticles at higher doses (≥10 μg/mL), resulting in a lower IC50 of the DDOX_ketal_-NPs1 nanoparticles. This probably resulted from the faster DOX release because of the looser aggregation structure of the DDOX_ketal_ dimeric prodrug in the DDOX_ketal_-NPs1 nanoparticles, which led to more micropores in the resultant nanoparticles. This also facilitated the diffusion of the H^+^ ions into the nanomedicine and the cutting of the ketal linker between the DOX units, as well as the diffusion of the released protonated DOX out of the nanomedicine.

## 3. Discussion

Recent work has explored the effect of the aggregation structure of the proposed DDOX_ketal_ dimeric prodrug on its acid-triggered DOX release and in vitro cytotoxicity on HepG2 cells. Similar to our previous works [21,23], the nanomedicine with the looser aggregation structure via fast precipitation showed a faster drug release, and subsequently a higher cytotoxicity, than the one with the compacter aggregation structure via slow self-assembly. These works revealed that the drug release performance could be also modulated by altering the aggregation structure beyond the molecular structure of the dimeric prodrug, resulting in better anti-tumor efficacy with the desired drug release rate.

Moreover, porous DDOX_ketal_ nanomedicines were obtained via both fast precipitation and slow self-assembly from the TEM observation in the present work. This was distinctly different from the results of the reported works [21,23], in which the microporous structure could not be observed with the TEM technique, although it could be revealed with the nitrogen adsorption/desorption technique [23]. With the looser aggregation structure via less π-π stacking, or the compacter aggregation structure via more π-π stacking, mesopores of around 8 nm could be seen in the DDOX_ketal_ nanomedicines. This demonstrated the effect of the linker between the drug molecules on the aggregation structure; specifically, a longer flexible linker might provide a diversified conformation of the dimeric prodrug, enabling its self-assembly via π-π stacking between the DOX units. Such findings indicated that the aggregation structure of the dimeric prodrug was determined by its molecular structure, and could also be used to regulate the drug release beyond its molecular structure.

## 4. Materials and Methods

### 4.1. Materials and Reagents

Doxorubicin hydrochloride (DOX⋅HCl, 99.4%) was bought from Beijing Huafang United Technology Co., Ltd. (Beijing, China). Triethylamine (TEA, >99.0%) was bought from Xilong Science Co., Ltd. (Shantou, China). 2,2-Dimethoxypropane (DMP, 98%) was bought from Adamas Co., Ltd. (Shanghai, China). *p*-Toluenesulfonic acid (TsOH, 99%) was purchased from Tianjin Guangxia Fine Chemical Research Institute (Tianjin, China). Other reagents and solvents were analytical grade and used as received. Double-distilled water was used throughout the experiments.

### 4.2. Analysis and Characterization

^1^H NMR spectra were recorded with a 400 MHz ^1^H NMR (JNM-ECS 400 M) in DMSO-d_6_. The UV-vis spectra and drug content were detected using a TU-1901 UV/vis spectrometer (Beijing Purkinje General Instrument Co., Ltd., Beijing, China) at 480 nm at room temperature. The fluorescent emission spectra were recorded by a Hitachi F-7500 fluorescence spectrometer (Hitachi High-Tech Corporation, Tokyo, Japan). Reversed-phase HPLC (RP-HPLC) analysis was performed on a Shimadzu HPLC system, equipped with a LC-20AP binary pump, an SPD-20A UV-Vis detector, and a Symmetry C18 column. The DDOX_ketal_ nanoparticles were observed with a transmission electron microscope (TEM, JEM-2100, Tokyo, Japan), and sampling with aqueous dispersion. The hydrodynamic diameter and distribution of the DDOX_ketal_ nanoparticles were measured using dynamic scattered light (DLS, BI-200SM, Brookhaven Instruments Corporation, Holtsville, NY, USA) in aqueous dispersion.

### 4.3. Synthesis of Dimeric Prodrug

The ketal-bridged doxorubicin dimeric prodrug (DDOX_ketal_) was synthesized by coupling two DOX molecules with an acid-sensitive acetone-based ketal conjugation [54]. Typically, DOX⋅HCl (58 mg, 0.10 mmol, 1.0 eq) was dissolved in 9 mL DMF. After adding 40 µL TEA, the solution was stirred overnight to de-acidify. Then, DMP (6 μL, 0.05 mmol, 0.5 eq) and TsOH (4 μg) were added into the solution. The reaction was conducted with stirring at 40 °C for 48 h in dark. The product was collected by dialyzing the resultant solution against water (molecular weight cutoff (MWCO) of 1 kDa) until the dialysate was colorless, and then centrifuged (10,000 rpm, 5 min) and finally dried in vacuum at 40 °C (yield: 78.6%).

### 4.4. DOX Content Measurement

The DOX content in the proposed ketal-bridged doxorubicin dimeric prodrug (DDOX_ketal_) was measured by determining the UV-vis absorption at 480 nm of its dimethyl sulfoxide (DMSO) solution on a TU-1901 UV-vis spectrometer and calculated with the calibration curve of DOX in DMSO solution (absorbance = 17.9623 * concentration (mg/mL) − 0.0010 (R^2^ = 0.9989)).

### 4.5. Fabrication of DDOX_ketal_ Nanoparticles

The DDOX_ketal_ nanoparticles were fabricated with two methods, as follows:

*DDOX_ketal_-NPs1 nanoparticles*: A quantity of 1.0 mL of DMF solution containing DDOX_ketal_ (1.0 mg/mL) was dropped into 10 mL of water at a rate of 1 drop per 10 s, with electromagnetic stirring at room temperature. The mixture was dialyzed against water (MWCO of 1 kDa) for 2 days, changing the dialysate every 4 h. Finally, the DDOX_ketal_-NPs1 nanoparticles were collected via lyophilization.

*DDOX_ketal_-NPs2 nanoparticles*: A quantity of 10 mL of water was dropped into 1.0 mL of DMF solution containing DDOX_ketal_ (1.0 mg/mL) at a rate of 1 drop per 10 s, with electromagnetic stirring at room temperature. The mixture was dialyzed against water (MWCO of 1 kDa) for 2 days, changing the dialysate every 4 h. Finally, the DDOX_ketal_-NPs1 nanoparticles were collected via lyophilization.

### 4.6. Acid-Triggered Drug Release

The DDOX_ketal_ nanoparticles (1.0 mg) were dispersed in 10 mL of release media with different pH values, pH 7.4 PBS and pH 5.0 ABS. The dispersion was dialyzed (MWCO of 1 kDa) in 140 mL of the corresponding releasing medium in an IS-RSD3 incubation shaker at 37 °C. At certain time intervals, 5.0 mL of the dialysate was taken out to measure the DOX concentration on a UV-vis spectrometer at 480 nm, with the calibration curves of DOX in the corresponding releasing medium (pH 7.4 PBS: absorbance = 16.2986 * concentration (mg/mL) + 0.0043 (R^2^ = 0.9980); pH 5.0 ABS: absorbance = 16.6109 * concentration (mmol/mL) + 0.0056 (R^2^ = 0.9976)), and 5.0 mL of the fresh buffer solution was added to maintain constant volume. The cumulative release was expressed as the total percentage of drug molecule released through the dialysis membrane over time.

### 4.7. In Vitro Cellular Experiments

After the HepG2 cells were incubated with 15 µg/mL of DDOX_ketal_ nanoparticles (DDOX_ketal_-NPs2 nanoparticles and DDOX_ketal_-NPs2 nanoparticles) for 24 h, they were fixed with paraformaldehyde and washed twice with pH 7.4 PBS; then, the nuclei were stained with DAPI and washed twice with pH 7.4 PBS. The HepG2 cells were analyzed on an inverted fluorescence microscope (OLYMPUS, IX71) (DAPI at 405 nm and DOX at 480 nm).

As for the in vitro cytotoxicity, the HepG2 cells were incubated with DDOX_ketal_-NPs1 nanoparticles, DDOX_ketal_-NPs2 nanoparticles, or free DOX at various DOX equivalent doses for 48 h. The cell viability was assessed with an MTT assay, using the Enzyme-linked Immunosorbent Assay Appliance at 490 nm.

## 5. Conclusions

In summary, an acid-triggered dimeric prodrug of doxorubicin (DOX) with a high drug content near the pure drug was synthesized by conjugating two DOX molecules with an acid-labile ketal linker. Different aggregation structures of the proposed DDOX_ketal_-based dimeric prodrug were obtained by two fabrication approaches of fast precipitation and slow self-assembly, as revealed by the UV-vis and fluorescence analysis. With a similar spherical shape and D_h_, the DDOX_ketal_-NPs1 nanoparticles via fast precipitation showed a faster acid-triggered DOX release and higher in vitro cytotoxicity on the HepG2 cells at higher doses, than the DDOX_ketal_-NPs2 nanoparticles via slow self-assembly. The results demonstrated that the drug release could be efficiently regulated by altering the aggregation structure of the dimeric prodrug, due to the different degrees of π-π stacking interaction between the DOX units. Moreover, it was also found that the aggregation structure of the dimeric prodrug was determined by its molecular structure, by comparison with the effect of the aggregation structure of the other dimeric prodrug. So, it could be concluded that the DOX release and in vitro cytotoxicity of the dimeric prodrugs could be regulated by altering either their molecular structure or their aggregation structure. As a result, the desired drug release behavior is expected, with higher antitumor efficacy, in future tumor treatment.

## Data Availability

The raw data supporting the conclusions of this article will be made available by the authors on request.
